# Clinical Significance of Velamentous Cord Insertion Prenatally Diagnosed in Twin Pregnancy

**DOI:** 10.3390/jcm10040572

**Published:** 2021-02-03

**Authors:** Hyun-Mi Lee, SiWon Lee, Min-Kyung Park, You Jung Han, Moon Young Kim, Hye Yeon Boo, Jin Hoon Chung

**Affiliations:** 1 Department of Obstetrics and Gynecology, CHA Ilsan Medical Center, CHA University, Goyang 10414, Korea; hmlee984415@gmail.com (H.-M.L.); lrhnb4@gmail.com (H.Y.B.); 2 Department of Obstetrics and Gynecology, Mount Sinai Medical Center, Miami Beach, FL 33109, USA; c1loveya@gmail.com; 3 Department of Obstetrics and Gynecology, Yonsei University College of Medicine, Seoul 03722, Korea; mk0801@naver.com; 4 Department of Obstetrics and Gynecology, CHA Gangnam Medical Center, CHA University, Seoul 06135, Korea; hanyj1978@gmail.com (Y.J.H.); mykimdr@gmail.com (M.Y.K.); 5 Department of Obstetrics and Gynecology, University of Ulsan College of Medicine, Asan Medical Center, Seoul 05505, Korea

**Keywords:** velamentous cord insertion, twin, perinatal outcomes, pregnancy outcomes, chorionicity, twin specific complications

## Abstract

Background: The purpose of this study was to evaluate the prevalence of velamentous cord insertion (VCI) and the actual association between pathologically confirmed VCI and perinatal outcomes in twins based on the chorionicity. Methods: All twin pregnancies that received prenatal care at a specialty clinic for multiple pregnancies, from less than 12 weeks of gestation until delivery in a single institution between 2015 and 2018 were included in this retrospective cohort study. Results: A total of 941 twins were included in the study. The prevalence of VCI in dichorionic (DC) twins and monochorionic diamniotic (MCDA) twins was 5.8% and 7.8%, respectively (*p* = 0.251). In all study population, the prevalence of vasa previa and placenta accreta spectrum was higher in VCI group than that of non-VCI group (*p* = 0.008 and 0.022). In MCDA twins with VCI, birth weight, 1 and 5-min Apgar score were lower than DC twins with VCI (*p* = 0.010, 0.002 and 0.000). There was no significant association between VCI and selective fetal growth restriction (*p* = 0.486), twin-to-twin transfusion syndrome (*p* = 0.400), and birth-weight discordance (>20% and >25%) (*p* = 0.378 and 0.161) in MCDA twins. Conclusion: There was no difference in the incidence of VCI in twins based on the chorionicity. Moreover, VCI was not a risk factor for adverse perinatal outcomes excepting vasa previa and placenta accreta spectrum, which had a high incidence in twins with VCI.

## 1. Introduction

Velamentous cord insertion (VCI), defined as an abnormal insertion of the umbilical vessels that insert into the fetal membranes before entering the placenta is one of the abnormal placental insertion findings of the umbilical cord.

The incidence of VCI has been reported to be 0.1–1.8% in all pregnancies [1]. An increased prevalence of VCI had been reported for twin pregnancies (1.6–40%) especially in monochorionic (MC) twin pregnancies, probably as a result of unequal sharing of the placentas [2,3].

A number of previous studies have reported that VCI is associated with adverse maternal and perinatal outcomes [1,4,5]. Likewise, VCI in MC twins has been proposed as a risk factor in the development of selective fetal growth restriction (sFGR), twin-to-twin transfusion syndrome (TTTS) and birth-weight (BW) discordance [4,6,7,8,9]. However, some studies were incongruent with the results and suggested that the adverse complications in VCI were possibly due to the selection bias and pointed out that these unique adverse outcomes in MC twins are likely the consequences of the vascular complications of the placental sharing rather than VCI [4,10,11].

Therefore, this study aimed to assess the prevalence of VCI in twin pregnancy and evaluate whether prenatally detected VCI was associated with adverse perinatal outcomes including unique complications of twin pregnancies based on the chorionicity.

## 2. Material and Methods

This study was a retrospective cohort study of twin pregnancies with known pregnancy outcomes and pathologically confirmed VCI in Cheil General Hospital and Women’s Healthcare Center between January 2015 and March 2018. The chorionicity was determined by the first trimester ultrasonography by the number of gestational sacs, yolk sacs, and fetuses. The monochorionic monoamniotic (MCMA) twin pregnancies with a high risk of perinatal complications and twin pregnancies with major congenital anomaly or aneuploidy were excluded. Ethical approval of the study was obtained from the institutional review board (CGH-IRB-2018-37). The need to obtain informed consent was waived.

**Figure 1:**
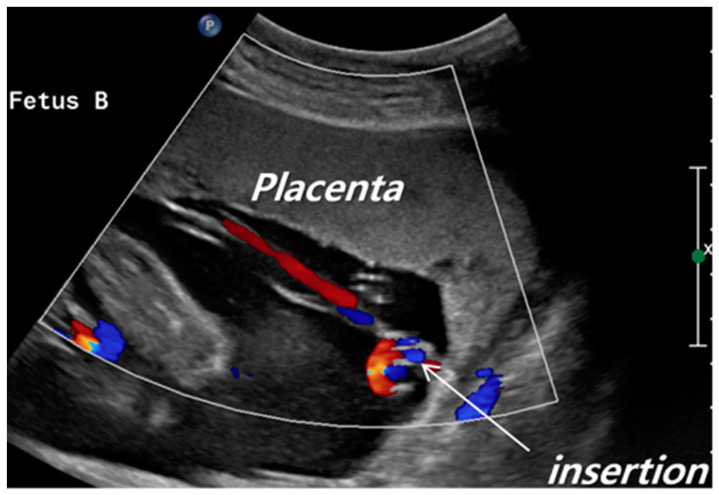
Velamentous cord insertion demonstrated with color Doppler imaging in the mid-trimester.

**Figure 2:**
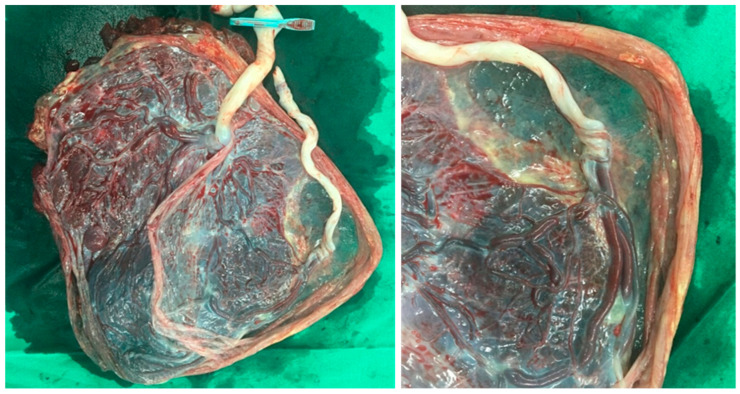
Photograph showing the placenta with a velamentous insertion of the umbilical cord in monochorionic twin pregnancy.

Placenta cord insertion site was categorized into two groups: VCI and non-VCI. VCI was determined during the anatomy ultrasound by a maternal fetal medicine (MFM) specialist using high-resolution sonography equipments (Voluson E8 (GE Healthcare, Milwaukee, WI, USA), EPIQ5 (Philips, Amsterdam, Netherlands)). As part of this examination, MFM specialist evaluated the placental umbilical cord insertion site using the appropriate magnification and settings. After identifying the umbilical cord, MFM specialist followed it until it reached the placental surface. The insertion site was further confirmed with color flow imaging if necessary. The VCI was diagnosed when the umbilical cord was attached to the placental membranes instead of the placental body. When VCI was detected, the patient underwent sonography again two weeks later by another MFM specialist. The MFM specialist performed either a transabdominal or transvaginal scan (Figure 1) [12]. The VCI group was defined as VCI in one or both of the fetuses. The chorionicity was determined by assessing the number of the gestational sac, yolk sac, and the shape of intertwin membranes on the first trimester ultrasound [13]. We confirmed the VCI and the chorionicity by postpartum pathological examination of the placenta (Figure 2).

Clinical significance of VCI in twin pregnancy was evaluated between VCI group and non-VCI group based on the chorionicity. Pregnancy and neonatal outcomes including preeclampsia, gestational diabetes mellitus (GDM), preterm premature rupture of membranes (PPROM), placenta previa, vasa previa, placenta accreta spectrum (PAS) [14], postpartum hemorrhage, BW discordance of more than 20% and 25%, BW of the neonates, TTTS, sFGR, 1 and 5-min Apgar score, and neonatal intensive care unit (NICU) admission were analyzed between the groups.

BW discordance was calculated by subtracting the smaller twin’s weight from the larger twin’s weight and then dividing by the larger twin’s weight, expressed as a percentage. sFGR was defined differently in MC and DC twin pregnancies. sFGR in MC twin pregnancy was diagnosed when the BW of one of the twins was below the 10th centile for gestational age and there was at least a 25% difference in estimated body weight between the fetuses [15]. We defined sFGR in DC twin pregnancy as an estimated fetal weight (EFW) < 10th centile [16]. Diagnosis of TTTS was made according to the established criteria, MC twin and discordant amniotic fluid volume (AFV) (MVP > 8 cm in the recipient twin, MVP < 2 cm in the donor twin) [17].

Statistical analyses were performed using the software SPSS for windows version 17.0 (SPSS, Inc., Chicago, IL, USA). The Chi-square test was used to assess the association between the fetal level data aggregated and pair level data with VCI. All significance tests were two-tailed. A *p*-value of less than 0.05 was considered statistically significant for all analyses.

## 3. Results

A total of 981 twin pregnancies were identified during the study period. After excluding 40 pregnancies with major congenital anomalies (*n* = 7), chromosomal abnormalities (*n* = 5), MCMA twins (*n* = 5), or lost to follow-up (*n* = 23), 941 twins (DC, *n* = 788 and MCDA, *n* = 153) were enrolled.

A total 58 cases (6.2%) of VCIs were diagnosed by ultrasound, including 46 cases (5.8%) of DC twin pregnancies and 12 cases (7.8%) of MCDA twin pregnancies. There was no statistically significant difference in the prevalence of VCI between DC and MCDA twin pregnancies (*p* = 0.251) in our study.

Maternal characteristics and perinatal outcomes of the twin pregnancies in VCI group compared to non-VCI groups are presented in Table 1. The two groups did not differ regarding maternal age at delivery (*p* = 0.128), pregnancies conceived via assisted reproductive technique (ART) (*p* = 0.969), gestational age at delivery (*p* = 0.077), preeclampsia (*p* = 0.395), GDM (*p* = 0.643), PPROM (*p* = 0.466), BW discordance (>20% and >25%) (*p* = 0.689 and 0.108), and sFGR (*p* = 0.934). VCI-group showed significantly higher rates of vasa previa (*p* = 0.008) and PAS (*p* = 0.022) compared to non-VCI group. All three cases of PAS were abnormally adherent placenta (Grade 1) [14]. There was no difference in neonatal outcomes including birth-weight, 1 and 5-min Apgar score, and the rate of NICU admission between VCI and non-VCI groups.

Table 2 shows the maternal characteristics, pregnancy, and neonatal outcomes in VCI group based on the chorionicity. There were no differences in maternal characteristics regarding maternal age at delivery (*p* = 0.325), gestational age at delivery (*p* = 0.078). No other differences in pregnancy outcomes were observed in twins with VCI based on the chorionicity. However, lower BW (*p* = 0.010), and lower 1and 5-min Apgar score (*p* = 0.002 and 0.000) were noted in MCDA twin pregnancy group compared to DC twin pregnancy group, despite no difference in gestational age at delivery.

When DC twin pregnancies were subdivided into VCI and non-VCI groups (Table 3), the two groups did not differ regarding maternal age at delivery (*p* = 0.106), gestational age at delivery (*p* = 0.196), preeclampsia (*p* = 0.714), GDM (*p* = 0.876), PPROM (*p* = 0.693), BW discordance (>20% and >25%) (*p* = 0.582 and 0.897), sFGR (*p* = 0.657), and neonatal outcomes including birth-weight, 1 and 5-min Apgar score, and the rate of NICU admission. Notably, vasa previa and PAS were more common in VCI than non-VCI groups in DC twin pregnancies (*p* = 0.018 and *p* = 0.018).

Table 4 presents the comparison of pregnancy outcomes in VCI and non-VCI group in MCDA twin pregnancies. There was no significant association between VCI and sFGR (*p* = 0.486), TTTS (*p* = 0.400), and BW discordance (>20% and >25%) (*p* = 0.378 and 0.161) in MCDA twin pregnancies.

## 4. Discussion

We evaluated the prevalence and associated adverse perinatal outcomes of VCI in twin pregnancies based on the chorionicity. The incidence of VCI in total twin pregnancies was 6.2% and the incidence of VCI in DC and MCDA twin pregnancies were 5.8% and 7.8%, respectively. The incidence of VCI in our study was lower compared to the previous studies [1,4,18]. Moreover, no significant difference in prevalence was noted between DC and MCDA twin pregnancies (*p* = 0.251) in our study.

Another notable finding of this study was that when VCI and non-VCI groups were compared based on the chorionicity, VCI group did not significantly associate with adverse pregnancy outcomes. Furthermore, VCI was not associated with twin specific complications including sFGR, TTTS, and BW discordance in MCDA twin pregnancies.

Many previous studies described the association between VCI and adverse pregnancy/ perinatal outcomes [1,4,6,7,8]. VCI was associated with an increased risk of fetal growth restriction, preterm labor, low Apgar scores, placental abruption, and fetal and neonatal death [5,18,19,20]. In MCDA twin pregnancy, when unequal sharing of the placental territory is present, the higher association between VCI and adverse outcomes such as sFGR, TTTS, and BW discordance had been described [1,3,6,7,8,21,22]. However, several studies with relatively large sample sizes came to a contradictory conclusion about the association between abnormal cord insertion in MCDA twin pregnancies and BW discordance/sFGR [9,23], and several studies also demonstrated no significant impact of VCI on TTTS [6,22,23]. Similarly, we did not observe significant differences in neonatal outcomes between VCI and non-VCI groups including BW, Apgar scores at 1 and 5-min, and NICU admission rates in twin pregnancies. Our results are consistent with the previous studies [9,23], which demonstrated no association between VCI and the risk of sFGR and BW discordance in MCDA twins. Furthermore, in our study, VCI group in MCDA twin pregnancy did not show association with TTTS, even if we included 2 cases with VCI transferred to a tertiary center for fetoscopic laser surgery. In particular, we included twin pregnancies with VCI only, unlike the previous study, which included marginal cord insertion and VCI, and found an association between abnormal cord insertion and sFGR, TTTS, and BW discordance in MCDA twins [24].

In MCDA twin pregnancies with VCI, birth-weight, may be a major factor of neonatal mortality, and Apgar scores at 1 and 5-min were lower than DC twins with VCI. This may be due to MC twin pregnancy’s natural characteristics, which is unequal sharing of the placental territory, not VCI, considering that there was no difference in the gestational age at delivery between MC and DC twin pregnancies with VCI.

In this study, twin pregnancies with VCI demonstrated higher incidence of vasa previa and PAS than those with non-VCI. Although, previous studies reported a higher risk of vasa previa in multiple pregnancies with VCI [1,4,25], our study showed no difference in the prevalence of vasa previa when analyzed based on the chorionicity.

Strengths of the study include that only twin pregnancies who underwent consistent prenatal care with MFM specializing in multiple pregnancies and delivered in the same institution were enrolled. Secondly, as this specialty clinic for multiple pregnancies included all multiple pregnancies with or without complications, unlike most tertiary care centers with referred complicated pregnancies, this study may reflect the general twin pregnancy population. Thirdly, in our study, compared to previous studies conducted in tertiary referral center or multicenter [6,23], the sample size was not small when considering that the study was conducted in a single institution. The main limitation of the study was that this was a retrospective study, and TTTS of stage II or higher had to be sent to a tertiary center, because fetoscopic laser coagulation was not performed in this institution, so the outcomes related to this were excluded from the analysis. However, as mentioned above, there was no association between VCI and TTTS, even when stage II or higher cases of TTTS were included. Instead, it was considered that selection bias would be small because it was targeted to twin pregnancies who had been prenatal care at the institution since the early pregnancy.

## 5. Conclusions

In conclusion, our study demonstrated that VCI in twin pregnancies was not a significant risk factor for adverse perinatal outcomes including twin-specific complications (sFGR, TTTS, BW discordance), although it increased the incidence of vasa previa and placenta accreta spectrum. The study results may help when counseling twin pregnancies diagnosed with VCI on prenatal ultrasound and avoid unnecessary medical interventions or concerns. In additions, meticulous follow-up will be required for the presence or absence of vasa previa to determine the timing and mode of delivery.

## Figures and Tables

**Table 1:** Comparison of maternal characteristics, pregnancy outcomes, and neonatal outcomes in twin pregnancies with or without velamentous cord insertion (*n* = 941).

	Velamentous	Non-Velamentous	*p*
	(*n* = 58)	(*n* = 883)	
Maternal characteristics			
Maternal age at delivery (years)	34.7 ± 3.3	35.3 ± 2.7	0.128
Pregnancy conceived via ART	48 (82.8%)	733 (83.0%)	0.969
GA at delivery (weeks)	36.0 ± 2.1	36.5 ± 1.4	0.077
Gravidity	1.7 ± 1.2	1.7 ± 0.9	0.948
Nulliparous	46 (79.3%)	768 (87.0%)	0.202
MCDA	12 (20.7%)	141 (16.0%)	0.633
DCDA	46 (79.3%)	742 (84.0%)	0.633
Pregnancy outcomes			
Preeclampsia	5 (8.6%)	115 (13.0%)	0.395
GDM	4 (6.9%)	79 (8.9%)	0.643
PPROM	10 (17.2%)	115 (13.0%)	0.466
Placenta previa	3 (5.2%)	26 (2.9%)	0.491
Vasa previa	4 (6.9%)	0	0.008
PAS	3 (5.2%)	0	0.022
Postpartum hemorrhage	28 (48.3%)	353 (40.0%)	0.311
PTB < 37 weeks	26 (44.8%)	331 (37.5%)	0.264
PTB < 34 weeks	6 (10.3%)	60 (6.8%)	0.305
Birth-weight discordance ≥ 20%	12 (20.7 %)	164 (18.6%)	0.689
Birth-weight discordance ≥ 25%	6 (10.3%)	47 (5.3%)	0.108
sFGR	10 (17.2%)	156 (17.7%)	0.934
Neonatal outcomes	*n* = 116 fetuses	*n* = 1766 fetuses	
Birth weight (g)	2418 ± 499	2377 ± 371	0.408
1-min Apgar Score	7.3 ± 0.86	7.4 ± 0.76	0.293
5-min Apgar Score	8.4 ± 0.65	8.4 ± 0.67	0.661
NICU admission	51 (44.0%)	653 (37%)	0.222

ART, assisted reproductive technology; GA, gestational age; MCDA, monochorionic diamniotic twins; DCDA, dichorionic diamniotic twins; GDM, gestational diabetes mellitus; PPROM, preterm premature rupture of membranes; PAS, placenta accreta spectrum; PTB, preterm birth; sFGR, selective fetal growth restriction; NICU, neonatal intensive care unit.

**Table 2:** Comparison of maternal characteristics and perinatal outcomes in twins with velamentous cord insertion according to chorionicity (*n* = 58).

	MC (*n* = 12)	DC (*n* = 46)	*p*
Maternal characteristics			
Maternal age at delivery (years)	33.8 ± 3.0	35.0 ± 3.3	0.325
Pregnancy conceived via ART	2 (16.7%)	46 (100%)	0
GA at delivery (weeks)	35.0 ± 2.5	36.2 ± 1.9	0.078
Pregnancy outcomes			
Preeclampsia	1 (8.3%)	4 (8.7%)	0.968
GDM	0	4(8.7%)	0.29
PPROM	2 (16.7%)	8 (17.4%)	0.953
Placenta previa	0	3 (6.5%)	0.364
Vasa previa	1 (8.3%)	3 (6.5%)	0.825
PAS	0	3 (6.5%)	0.364
Postpartum hemorrhage	6 (50.0%)	22 (47.8%)	0.893
PTB < 37 weeks	8 (66.7%)	18 (39.1%)	0.088
PTB < 34 weeks	2 (16.7%)	4 (8.7%)	0.419
Birth-weight discordance ≥ 20%	4 (33.3%)	8 (17.4%)	0.549
Birth-weight discordance ≥ 25%	3 (25.0%)	3 (6.5%)	0.061
sFGR	3 (25.0%)	7 (15.2%)	0.424
Neonatal outcomes	*n* = 24 fetuses	*n* = 92 fetuses	
Birth weight (g)	2186 ± 485	2479 ± 487	0.01
1-min Apgar Score	6.8 ± 0.9	7.4 ± 0.8	0.002
5-min Apgar Score	7.9 ± 0.6	8.5 ± 0.6	0
NICU admission	14 (58.3%)	37 (40.2%)	0.111

MC, monochorionic twins; DC, dichorionic twins; ART, assisted reproductive technology; GA, gestational age; GDM, gestational diabetes mellitus; PPROM, preterm premature rupture of membranes; PAS, placenta accreta spectrum; PTB, preterm birth; sFGR, selective fetal growth restriction; NICU, neonatal intensive care unit.

**Table 3:** Comparison of maternal characteristics and perinatal outcomes in dichorionic twin pregnancies according to velamentous cord insertion (*n* = 788).

	Velamentous	Non-velamentous	*p*
	(*n* = 46)	(*n* = 742)	
Maternal characteristics			
Age at delivery (years)	34.9 ± 3.3	35.2 ± 3.1	0.106
Pregnancy conceived via ART	46 (100%)	626 (95.1%)	0.133
GA at delivery (weeks)	36.2 ± 1.9	36.6 ± 1.4	0.196
Nulliparous, *n* (%)	36 (78.3%)	603 (91.6%)	0.195
Pregnancy outcomes			
Preeclampsia	4 (8.7%)	70 (10.6%)	0.714
GDM	4 (8.7%)	63 (9.6%)	0.876
PPROM	8 (17.4%)	94 (14.3%)	0.693
Placenta previa	3 (6.5%)	24 (3.6%)	0.443
Vasa previa	3 (6.5%)	0	0.018
PAS	3 (6.5%)	0	0.018
Postpartum hemorrhage	22 (47.8%)	274 (41.6%)	0.499
PTB < 37weeks	18 (39.1%)	247 (33.3%)	0.416
PTB < 34weeks	4 (8.7%)	46 (6.2%)	0.5
Birth-weight discordance ≥ 20%	8 (17.4%)	141 (21.4%)	0.582
Birth-weight discordance ≥ 25%	3 (6.5%)	39 (5.9%)	0.897
sFGR	7 (15.2%)	132 (17.8%)	0.657
Neonatal outcomes	*n* = 92 fetuses	*n* = 1484 fetuses	
Birth weight (g)	2479 ± 487	2408 ± 357	0.184
1-min Apgar Score	7.4 ± 0.8	7.4 ± 0.7	0.914
5-min Apgar Score	8.6 ± 0.6	8.5 ± 0.6	0.658
NICU admission	37 (40.2%)	485 (32.7%)	0.228

ART, assisted reproductive technology; GA, gestational age; GDM, gestational diabetes mellitus; PPROM, preterm premature rupture of membranes; PAS, placenta accreta spectrum; PTB, preterm birth; sFGR, selective fetal growth restriction; NICU, neonatal intensive care unit.

**Table 4:** Comparison of maternal characteristics and perinatal outcomes in monochorionic diamniotic twin pregnancies according to velamentous cord insertion (*n* = 153).

	Velamentous (*n* = 12)	Non-Velamentous (*n* = 141)	*p*
Maternal characteristics			
Age at delivery (years)	33.8 ± 3.0	34.5 ± 3.1	0.576
Pregnancy conceived via ART	2 (16.7%)	23 (16.3%)	0.887
GA at delivery (weeks)	35.0 ± 2.5	35.8 ± 1.8	0.323
Nulliparous, *n* (%)	8 (66.7%)	78 (55.3%)	0.820
Pregnancy outcomes			
Preeclampsia	1 (8.3%)	11 (7.8%)	0.254
GDM	0	8 (5.7%)	0.378
PPROM	2 (16.7%)	8 (5.7%)	0.378
Placenta previa	1 (8.3%)	0	0.240
Vasa previa	1 (8.3%)	0	0.240
PAS	0	0	-
Postpartum hemorrhage	6 (50.0%)	39 (27.7%)	0.315
PTB < 37weeks	8 (66.7%)	84 (59.6%)	0.630
PTB < 34weeks	2 (16.7%)	14 (9.9%)	0.464
Birth-weight discordance ≥ 20%	4 (33.3%)	23 (16.3%)	0.378
Birth-weight discordance ≥ 25%	3 (25.0%)	8 (5.7%)	0.161
sFGR	3 (25.0%)	24 (17.0%)	0.486
TTTS	0	7 (5.0%)	0.400
Neonatal outcomes	*n* = 24 fetuses	*n* = 282 fetuses	
Birth weight (g)	2186 ± 485	2214 ± 413	0.816
1-min Apgar Score	6.8 ± 0.9	7.2 ± 0.9	0.123
5-min Apgar Score	7.9 ± 0.6	8.1 ± 0.8	0.283
NICU admission	14 (58.3%)	148 (52.5%)	0.938

ART, assisted reproductive technology; GA, gestational age; GDM, gestational diabetes mellitus; PPROM, preterm premature rupture of membrane; TTTS, twin-to-twin transfusion syndrome; PAS, placenta accreta spectrum; PTB, preterm birth; sFGR, selective fetal growth restriction; NICU, neonatal intensive care unit.

## Data Availability

The data presented in this study are available on request from the corresponding author. The data are not publicly available due to privacy.
